# Stem cell therapy for white matter disorders: don’t forget the microenvironment!

**DOI:** 10.1007/s10545-016-9925-1

**Published:** 2016-03-21

**Authors:** Stephanie Dooves, Marjo S. van der Knaap, Vivi M. Heine

**Affiliations:** Department of Pediatrics/Child Neurology, VU University Medical Center, De Boelelaan 1117, 1081 HV Amsterdam, The Netherlands; Department of Functional Genomics, Center for Neurogenomics and Cognitive Research, VU University, Amsterdam, The Netherlands; Department of Complex Trait Genetics, Center for Neurogenomics and Cognitive Research, VU University, De Boelelaan 1085, 1081 HV Amsterdam, The Netherlands

## Abstract

White matter disorders (WMDs) are a major source of handicap at all ages. They often lead to progressive neurological dysfunction and early death. Although causes are highly diverse, WMDs share the property that glia (astrocytes and oligodendrocytes) are among the cells primarily affected, and that myelin is either not formed or lost. Many WMDs might benefit from cell replacement therapies. Successful preclinical studies in rodent models have already led to the first clinical trials in humans using glial or oligodendrocyte progenitor cells aiming at (re)myelination. However, myelin is usually not the only affected structure. Neurons, microglia, and astrocytes are often also affected and are all important partners in creating the right conditions for proper white matter repair. Composition of the extracellular environment is another factor to be considered. Cell transplantation therapies might therefore require inclusion of non-oligodendroglial cell types and target more than only myelin repair. WMD patients would likely benefit from multimodal therapy approaches involving stem cell transplantation and microenvironment-targeting strategies to alter the local environment to a more favorable state for cell replacement. Furthermore most proof-of-concept studies have been performed with human cells in rodent disease models. Since human glial cells show a larger regenerative capacity than their mouse counterparts in the host mouse brain, microenvironmental factors affecting white matter recovery might be overlooked in rodent studies. We would like to stress that cell replacement therapy is a highly promising therapeutic option for WMDs, but a receptive microenvironment is crucial.

“Leukoencephalopathies” or “white matter disorders” (WMDs) are all disorders that predominantly or exclusively affect the brain white matter. They can be inherited or acquired. Common acquired WMDs are multiple sclerosis (MS; incidence 1:1000) and periventricular leukomalacia (PVL; incidence 1:1800). The genetic leukoencephalopathies, collectively called the “leukodystrophies”, include several inborn errors of metabolism. They mainly affect children and often cause progressive white matter destruction and loss, leading to severe disabilities and early death. This group of disorders consists of many rare to extremely rare disorders, but as a group the incidence is unexpectedly high, estimated at ~1:7000 live births (Bonkowsky et al 2010). In patients with leukodystrophies the white matter of the central nervous system (CNS) is affected, with or without peripheral nervous system involvement, based on glial cell or myelin sheath abnormalities (Vanderver et al 2015). Better treatment is urgently needed for WMDs at all ages. Glia replacement therapies have great prospects for WMDs, especially for the leukodystrophies as these are caused by a genetic defect, which is not present in the transplanted cells.

White matter consists of myelin, oligodendrocytes, axons, astrocytes, and microglial cells. Myelin protects the health of axons and increases the speed and efficiency of neuronal conductance. In the CNS, the oligodendrocytes are responsible for myelin formation, while astrocytes have many homeostatic functions including supporting the formation and maintenance of myelin. In WMDs different cells or structures may be primarily or secondarily involved. Mutations in the proteolipid protein 1 (*PLP1*), a predominant myelin protein, are associated with X-linked Pelizaeus-Merzbacher disease (PMD; OMIM #312080) and affect the compactness of myelin and maturation of oligodendrocytes. Other WMDs are caused by mutations in genes predominantly expressed in astrocytes. A typical example is Alexander disease (OMIM #203450), a progressive leukodystrophy, caused by mutations in the *GFAP* gene (Messing et al 2010). Also, megalencephalic leukoencephalopathy with subcortical cysts (MLC; OMIM #604004) is caused by lack of the astrocyte-specific MLC1 protein, either through mutations in the *MLC1* gene (73 %) or in the *GLIALCAM* gene, encoding the MLC1 chaperone GlialCAM (20 %), which ensures proper localization at astrocytic endfeet (Dubey et al 2015). Autopsies of patients with WMDs not only reveal lack of myelin and astrogliosis, but also axonal and neuronal degeneration. Examples are the widespread affected axonal integrity in patients with 4H (hypomyelination, hypodontia, hypogonadotropic hypogonadism) leukodystrophy (Vanderver et al 2013) (OMIM #614381) and the neuroaxonal degeneration in patients with PMD (Garbern et al 2002). Microglial cell involvement is illustrated by MS where microglia activation contributes to inflammatory demyelination and neurodegeneration (Mahad et al 2015). Interestingly many WMDs are caused by genes which are ubiquitously expressed by all cells. Vanishing white matter disease (VWM; OMIM #603896) is one of the most prevalent genetic childhood WMDs, caused by mutations in any of the five subunits of eukaryotic translation initiation factor eIF2B. Although eIF2B is involved in all cells of our body, autopsies indicate predominant involvement of the oligodendrocytic and astrocytic cell types (Bugiani et al 2013). Recent studies confirm a major role for astrocyte dysfunction in VWM (Dooves et al 2016). Since WMDs show changes in the astrocyte and oligodendrocyte populations, and because their progenitors have migrative and plastic properties, they are attractive candidates for glial cell replacement therapy. Of note, white matter abnormalities have also been appreciated in a number of neurodegenerative diseases traditionally viewed as primary neuronal disorders. Examples are the toxicity of astrocytes in amyotrophic lateral sclerosis (Meyer et al 2014) and problems with white matter connectivity in schizophrenia (Landek-Salgado et al 2015). Because glia contributions are recognized, at least in a subset of neuronal disorders, glial-targeted therapy strategies for these disorders have been suggested as well.

In the design of cell replacement therapy, the complexity of white matter should be considered, as all components of the white matter microenvironment potentially impact the integrity of the white matter and the survival or functioning of (transplanted) cells. The microenvironment is defined as the local environment in the brain or in a specific brain region, and consists of (neural and non-neural) cells, the extracellular matrix (ECM), blood derived factors and factors provided by cells. The microenvironment is defined according to a certain point of reference, e.g., the microenvironment of white matter oligodendrocytes and myelin is everything in the local environment, potentially impacting these cells and the myelin. The composition of the microenvironment is often changed in disease. Since neurons, microglia, and astrocytes are all important partners in creating the right microenvironment for myelin maintenance and repair, cell transplantation strategies might require, at least in a subset of WMDs, inclusion of non-oligodendroglial cell types.

So, we argue that white matter repair therapies, next to glia replacement, should also involve strategies targeting the microenvironment to make it sufficiently receptive for transplanted cells.

A number of successful cell replacement therapies have been performed in rodent models of hypomyelinating diseases, suggesting the feasibility of such strategies in human subjects. The most commonly used model is the shiverer mouse, which has a major deletion in the myelin basic protein gene (*MBP*). The homozygous shiverer mutants present with a shivering phenotype, tonic seizures, and have a life span of 14–21 weeks depending on modulating factors like an immunodeficient background (Uchida et al 2012). Histology reveals myelin deficiency. It has proven to be possible to rescue the phenotype of immunodeficient shiverer mice by injecting human glial progenitor cells (Windrem et al 2008). This has led to the first early-phase clinical trial to test safety of neural stem cells and to identify proof of myelination after transplantation. Four male patients with an early-onset severe form of PMD were injected with clinical-grade human CNS stem cells (HuCNS-SCs). At a 1 year follow-up, the results assure safety and suggest preliminary efficacy (Gupta et al 2012). The authors report that patients showed stable to minor gains in neurological function after transplantation, and that the imaging studies give signals that could indicate myelination in transplanted regions. Although it is expected that the numbers of cells transplanted were too low to obtain considerable improvements, the authors suggested that immunosuppressive agents might also have inhibited myelination by donor cells. This underscores the importance of modulating the microenvironment into a receptive state in order to obtain successful cell replacement. This first clinical report encourages the continuation of investigation of cell replacement therapies to achieve white matter repair, with an initial focus on leukodystrophies.

Since factors secreted by astrocytes or microglia play an important role in oligodendrocyte functioning in both health and disease (Boulanger and Messier 2014), cell replacement might fail to remove adverse elements in the diseased host microenvironment threatening the success of myelin repair. For example, studies have shown that in MS lesion sites the inflammatory environment can inhibit neural progenitor cell survival (Giannakopoulou et al 2011), and that residing reactive astrocytes secrete growth factors like FGF2 and BMPs, and ECM components like hyaluronan that can inhibit oligodendrocyte maturation (Moore et al 2011). Globoid cell leukodystrophy, or Krabbe disease (OMIM #245200), is caused by deficiency of galactocerebrosidase (GALC; EC: 3.2.1.46). Deficiency of GALC leads to accumulation of galactosylceramide and its toxic derivative psychosine in oligodendrocytes and subsequently to a more generally increased psychosine level in the brain. Oligodendrocytes are sensitive to psychosine and die, but the increased psychosine levels also affect other cells like microglia. A cell replacement study with healthy oligodendrocyte progenitor cells, which are resistant to psychosine toxicity, most likely failed due to the die-off of donor cells the moment they matured into MBP-positive psychosine-sensitive oligodendrocytes (Kuai et al 2015). Furthermore, a recent study by Sekiya et al (2015), investigating the effects of transplantation of neuroblast cells after nerve injury, showed the importance of injection site of the transplant. The authors showed that cells injected on top of the damaged nerve were able to enter the damaged nerve tissue and cause functional recovery, while cells directly injected into the damaged area did not survive. These studies illustrate that the microenvironment can have a major impact on the success of cell replacement. Therefore modulation of the diseased microenvironment and specific localization of cells need careful considerations while designing cell transplantation therapies (Fig. 1).

**Fig. 1:**
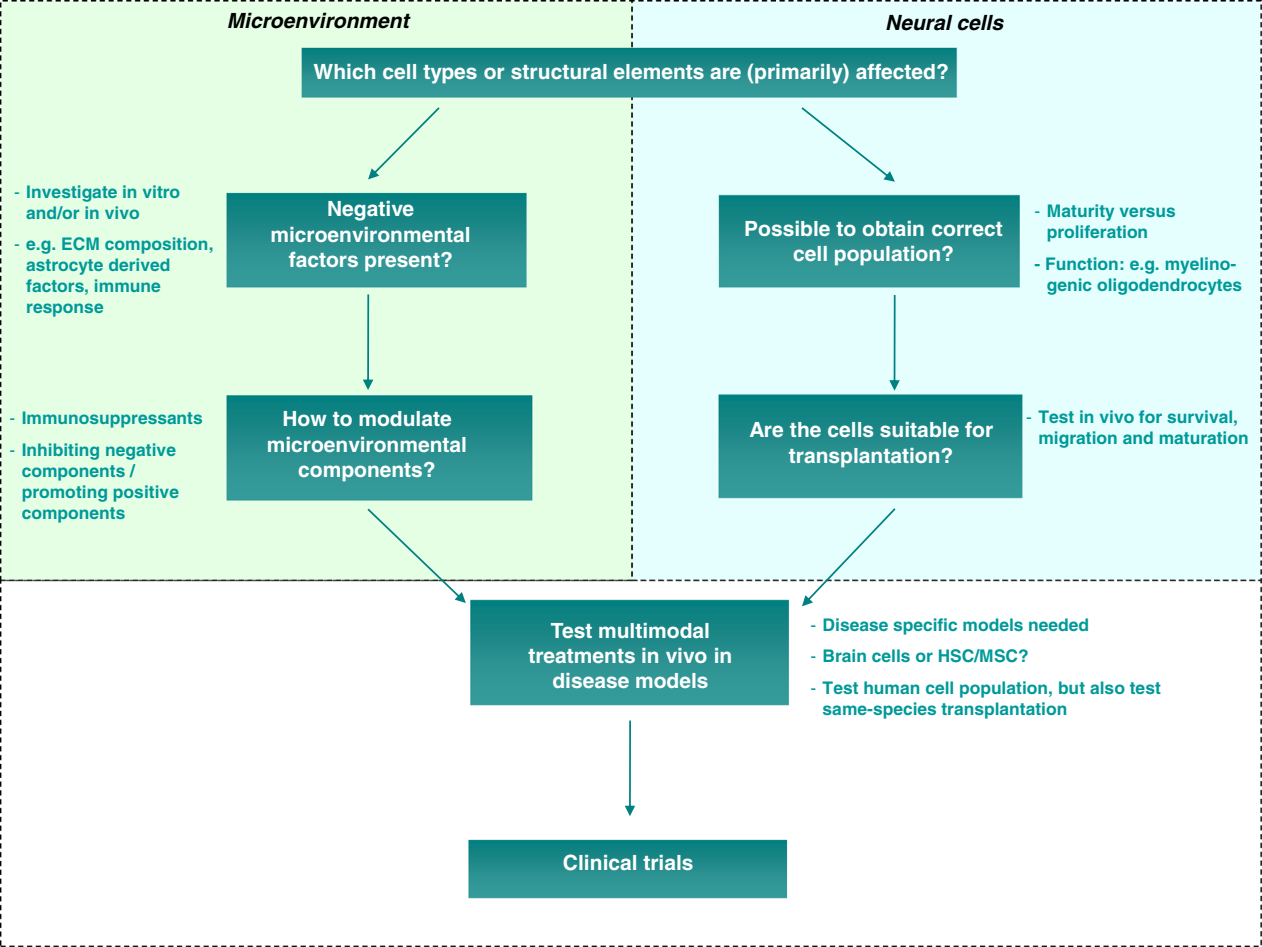
Considerations in the development of cell replacement therapy. To develop cell replacement therapy it is essential to consider the microenvironment that donor cells encounter after transplantation. Negative microenvironmental factors need to be considered (left side of the figure); these can be investigated in vitro using, e.g., co-cultures or conditioned medium experiments or in vivo by for example immunostainings for ECM components or RNA/protein analysis. If microenvironmental components are hypothesized to have a negative influence on donor cells, they need to be modulated for cell replacement therapy to be successful. The right side of the figure represents the cellular studies that are needed; is it possible to obtain or make the affected cell type in vitro, and will these cells survive and integrate after transplantation? Currently, most studies are focused on this part of therapy development. When appropriate cells and microenvironmental modulating strategies are developed, they can be combined in a multimodal therapy in disease-specific models. In this stage it is important to consider if brain cells are really needed or if HSC or MSC transplantation might provide a better option. Human cell populations need to be tested in vivo to asses their functionality, but it is also important to test same-species transplantation to see the efficacy of the cell replacement therapy without the phylogenic advantage of human donor cells

Cell transplantation is not always aimed at replacing affected cells, but practiced to modulate the microenvironment to improve glia maturation and white matter repair. This is shown by transplantation studies in the experimental autoimmune encephalomyelitis (EAE) animal models for MS, which demonstrated that the grafted cells did not participate directly in myelination, but instead released immunomodulatory and neurotrophic factors that improved the disease symptoms (Pluchino et al 2009). This is also illustrated by strategies involving non-neural cell types such as hematopoietic (HSC) or mesenchymal (MSC) stem cells. HSC transplantation has been used in clinics for over 30 years and is optional for some WMDs. After grafting, HSCs differentiate into monocytes, migrate throughout the body and become microglial cells in the brain. Although HSCs do not differentiate into oligodendrocytes, HSC-derived microglial cells can provide the missing enzymes to resident oligodendrocytes and can remove inhibitory myelin debris in the microenvironment, thereby slowing or halting disease progression. For example in patients with early-stage cerebral X-linked adrenoleukodystrophy (OMIM #300100) and metachromatic leukodystrophy (OMIM #604004) HSC transplantation improved the white matter abnormalities (Miller et al 2011; Boucher et al 2015). However, HSC transplantations have a significant risk in developing graft-versus-host disease. Since MSCs lack HLA class II antigens and therefore are resistant to graft-versus-host disease, MSC transplantation might provide a better treatment option than HSCs (Le Blanc et al 2003). MSCs, derived from bone marrow or umbilical cord blood, are shown to inhibit autoimmune attacks on the CNS, secrete neurotrophic factors, recruit oligodendrocytes and neural progenitor cells to lesion sites and/or modulate microglia activation to a more protective phenotype (Uccelli et al 2011). Although HSC or MSC transplantations have especially clear prospects for WMDs involving enzyme deficiencies or autoimmunity, they can also support general mechanisms involved in myelin repair.

An important part of the microenvironment is the ECM. The ECM is composed of fibrous proteins and glycosaminoglycans (GAGs) which are produced locally by different neural cell types and secreted into the extracellular space were they form a dense network and give structural support. The composition of the ECM is dynamic and is a source for molecular signals that influence many processes like development and neuronal signaling (Lau et al 2013). The ECM has a strong influence on the response of glial cells to brain damage and can promote or inhibit repair mechanisms. This is shown in MS, where altered expression of ECM proteins is thought to inhibit oligodendrocyte progenitor cell maturation and myelin repair (Lau et al 2013). It is expected that in many WMDs the ECM is altered as a consequence of glial dysfunction. Recent studies show that the ECM component hyaluronan is increased in the brains of VWM patients (Bugiani et al 2013) and Tenascin-C production is increased in the brains of patients with Krabbe disease (Claycomb et al 2014). As the ECM can be crucial for the survival, integration, and maturation of cells after transplantation, we need better understanding of WMD-specific changes in the ECM. Enzymatic clearance of negative ECM components has been shown to improve recovery after spinal cord injury in rodent models (Lau et al 2013). For diseases that involve multifocal lesions, diminishing the inhibitory ECM components already at the production stage might be more favorable, as shown in the lysolecithin mouse model of demyelination (Lau et al 2013). Other potential strategies can involve promotion of positive ECM components or inhibition of receptors for ECM components, although these await in vivo testing. ECM targeting interventions will likely be part of future therapy approaches for WMDs.

It is expected that many WMDs will benefit from combinational therapies involving stem cell transplantation and microenvironment targeting strategies. Several studies using either in vivo or in vitro methods addressed microenvironmental factors in WMDs. For spinal cord injury, in vivo modulation of the injection site environment using bioscaffolds containing growth factors showed increased survival of donor cells and functional recovery compared to transplants of cells alone or cells on bioscaffolds without growth factors (Johnson et al 2010). Others suggested to push resident microglia to a remyelination-supportive phenotype in MS (Olah et al 2012). A large range of co-cultures or conditioned medium experiments which model aspects of oligodendrocyte survival and functioning, including the influence of other cell types, has recently been reviewed by (Barateiro and Fernandes 2014). In vitro assays give the possibility to test positive and negative environmental factors and suggested interventions in higher throughput than in vivo studies. Prospects for multimodal therapies have recently been explored for Krabbe disease (Ricca et al 2015). The authors treated a mouse model for Krabbe disease with either neural stem cell transplantation or intracerebral gene therapy, followed by HSC transplantation. Control mice were untreated or received only one of the treatments. Both neural stem cell transplantation and intracerebral gene therapy decreased the levels of toxic psychosine in the brain, which allowed the transplanted HSCs more time to integrate. Although all treated mice showed improvement over the untreated mice, those that received multimodal treatment showed the largest improvement.

The existence of representative disease models to test the transplantation and modulations of the microenvironment in vivo will be crucial. Many of the current proof-of-concept studies for cell replacement therapies for leukodystrophies are performed in animal models modeling myelin defects (e.g., Shiverer mouse), but not a human disorder. To successfully design transplantation strategies tailored to each disease, we need animal models which representatively mimic human disease. Recently we have developed mouse models representative for the leukodystrophies MLC (Dubey et al 2015) and VWM (Dooves et al 2016).

Another critical consideration involving the microenvironment is the performance of same (allo-) or different species (xeno-) transplantations. Human glial cells can be markedly different from their rodent counterparts, e.g., astrocytes. Nine months after transplantation of human glial progenitor cells (GPCs) in both shiverer and WT mouse models, all GPCs in the mouse are of human origin (Windrem et al 2014). By contrast, WT mouse GPCs injected in the brains of shiverer mice do not have the ability to outcompete host GPCs and do not show the same migration capacity as the human GPCs (Windrem et al 2014). If human glial cells have this large phylogenetic advantage over rodent cells, it might also make them less susceptible to factors released in the (diseased) microenvironment. The number of studies using rodent cells for transplantation is small, and although most of those studies show integration and myelination of donor cells, very few show a rescue of the phenotype (Kuai et al 2015; Giannakopoulou et al 2011; Plemel et al 2011). Therefore, in order to have a more accurate estimation about the prospects for cell replacement therapy, it would be best to test the improvement of the phenotype with both rodent and human cells, to avoid overlooking microenvironmental factors in preclinical studies.

In conclusion, WMDs show white matter changes involving myelin, oligodendrocytes, astrocytes, microglia, and/or axons. Nevertheless, a large group of WMDs will benefit from transplantation of a competent glial progenitor population that is capable of migration, successful integration, and differentiation into astrocytes and/or myelinogenic oligodendrocytes. Over time many viable populations of human glial progenitor cells have been developed, either derived from fetal tissue or from patient-derived pluripotent stem cells. The development of new representative animal models for WMDs will facilitate the investigation of environmental factors of importance to the development of optimal transplantation strategies for every specific disease. When the microenvironment is taken into account, cell transplantation is a very promising therapeutic option for WMD!
